# Supplementary material for: An aqueous electrolyte densified by perovskite SrTiO3 enabling high-voltage zinc-ion batteries
Source: Nat Commun. 2023 Aug 17;14:4981. doi: 10.1038/s41467-023-40462-z (PMC10435537; doi:10.1038/s41467-023-40462-z)
Supplement: Supplementary file 1 — Supplementary Information [file 41467_2023_40462_MOESM1_ESM.pdf]

Supplementary Information

An aqueous electrolyte densified by perovskite $SrTiO_3$ enabling high-voltage zinc-ion batteries

*Rongyu Deng[1], Zhenjiang He[1], Fulu Chu[1], Jie Lei[1], Yi Cheng[1], You Zhou[1] and Feixiang Wu[1]**

[1] School of Metallurgy and Environment, Engineering Research Center of the Ministry of Education for Advanced Battery Materials, Hunan Provincial Key Laboratory of Nonferrous Value-added Metallurgy, Central South University, Changsha 410083, PR China

* Corresponding author: F. Wu, (feixiang.wu@csu.edu.cn)

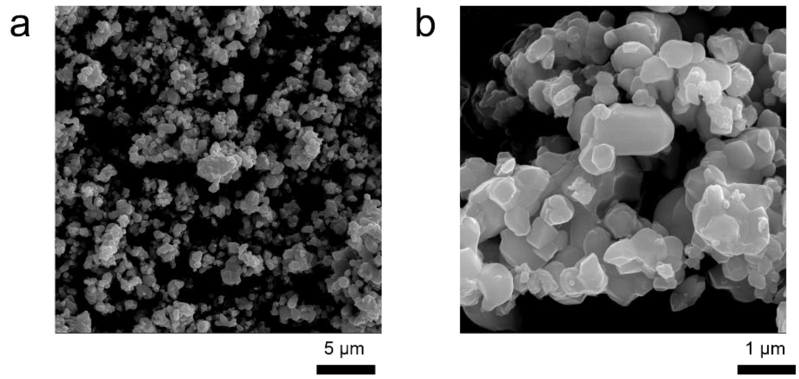

Supplementary Fig. S1 | The SEM images of $SrTiO_3$ particles. **a** at a small multiple. **b** the corresponding enlarged images clearly shows the sizes of the particles.

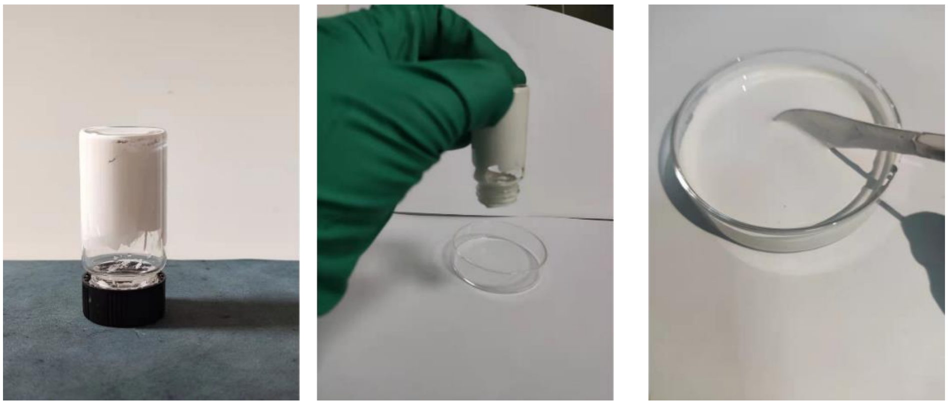

Supplementary Fig. S2. The optical photos of the densified electrolyte. The densified electrolyte is less fluid, so it is difficult to flow out of the bottle.

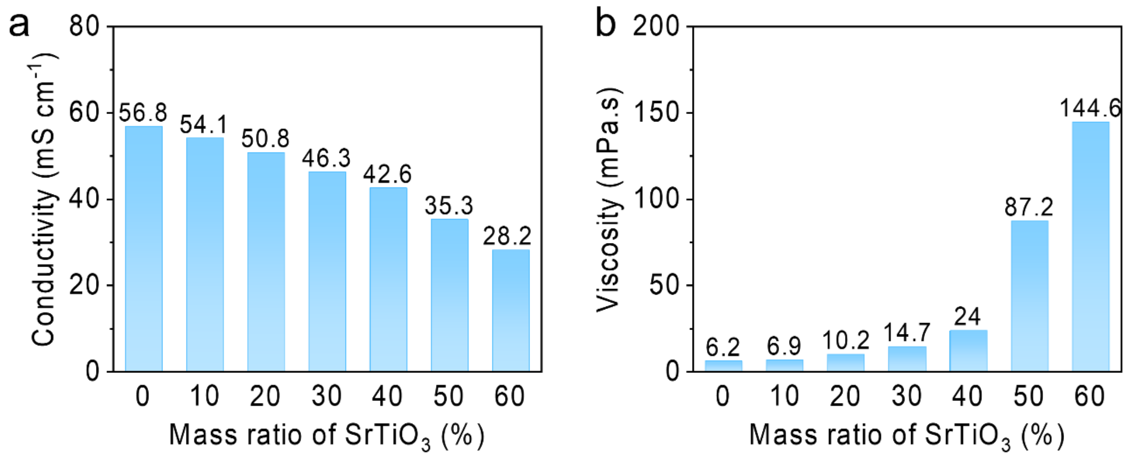

Supplementary Fig. S3 | The conductivity and viscosity of the densified electrolyte. **a** The conductivities of electrolytes with different $SrTiO_3$ contents. **b** their corresponding viscosities tested on a rotational rheometer at a shear rate of 1000 s^{-1}.

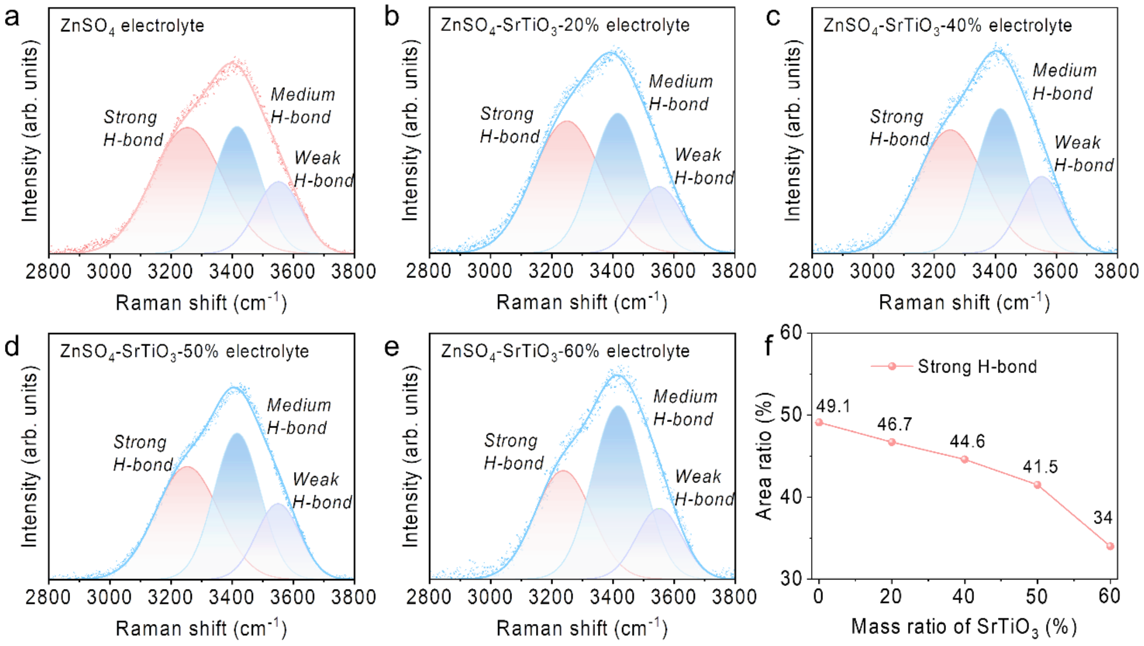

Supplementary Fig. S4 | Raman spectra with fit peaks of different electrolytes: **a** $ZnSO_4$ electrolyte, **b** $ZnSO_4$ electrolyte with 20 wt.% $SrTiO_3$, **c** $ZnSO_4$ electrolyte with 40 wt.% $SrTiO_3$, d $ZnSO_4$ electrolyte with 50 wt.% $SrTiO_3$, **e** $ZnSO_4$ electrolyte with 60 wt.% $SrTiO_3$; **f** The ratio of strong H-bond area of electrolytes with various $SrTiO_3$ contents.

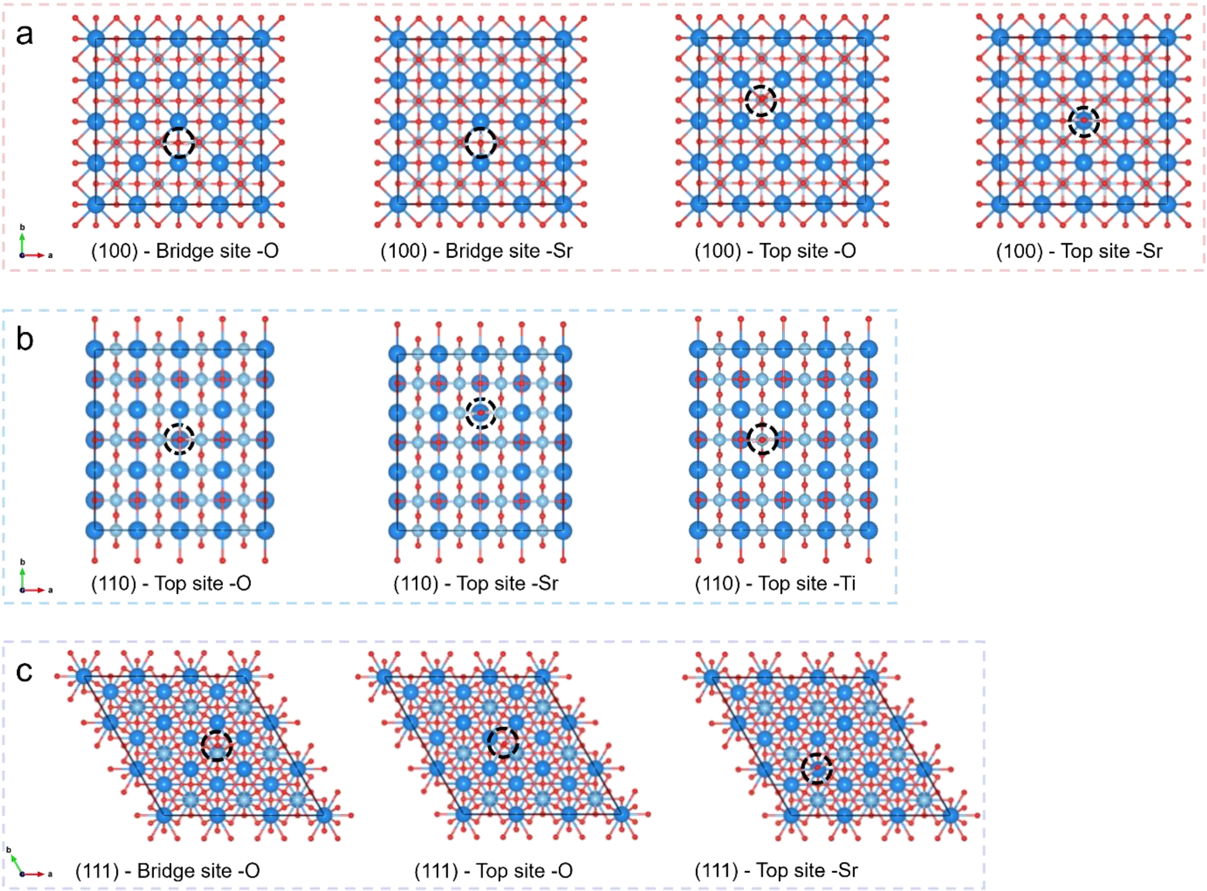

Supplementary Fig. S5 | The Top view of geometrical configurations of H_2O adsorbed on the various $SrTiO_3$ planes (the dark blue ball represents the Sr atom, the indigo ball is the Ti atom, the red ball is the O atom, and H_2O molecules are in the black rings). **a** (100) plane, **b** (110) plane and **c** (111) plane.

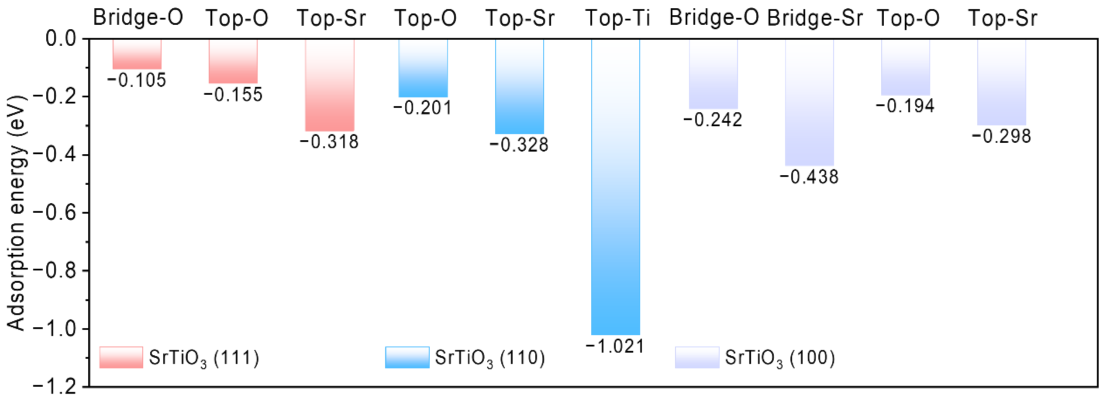

Supplementary Fig. S6 | The adsorption energy of H_2O adsorbed on the various $SrTiO_3$ (110) plane.

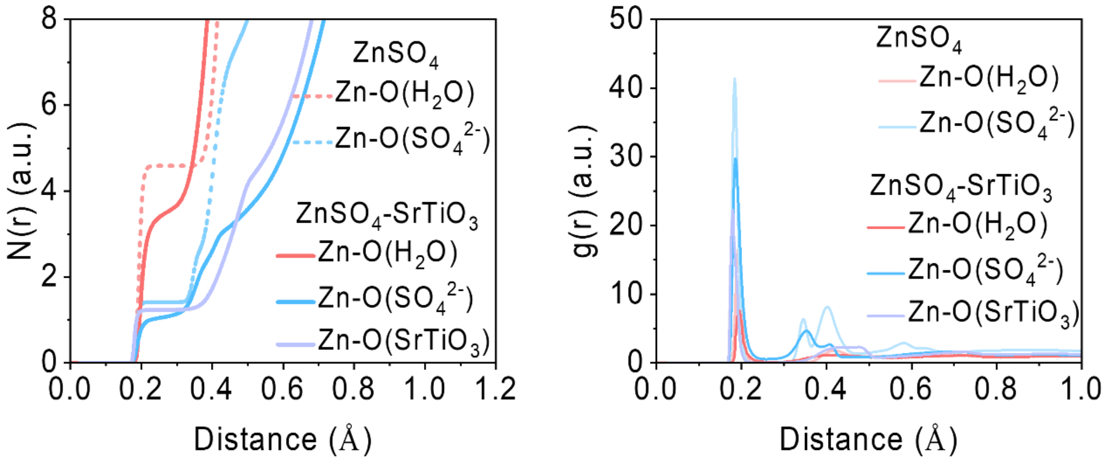

Supplementary Fig. S7 | The solvation structure of the densified electrolyte.

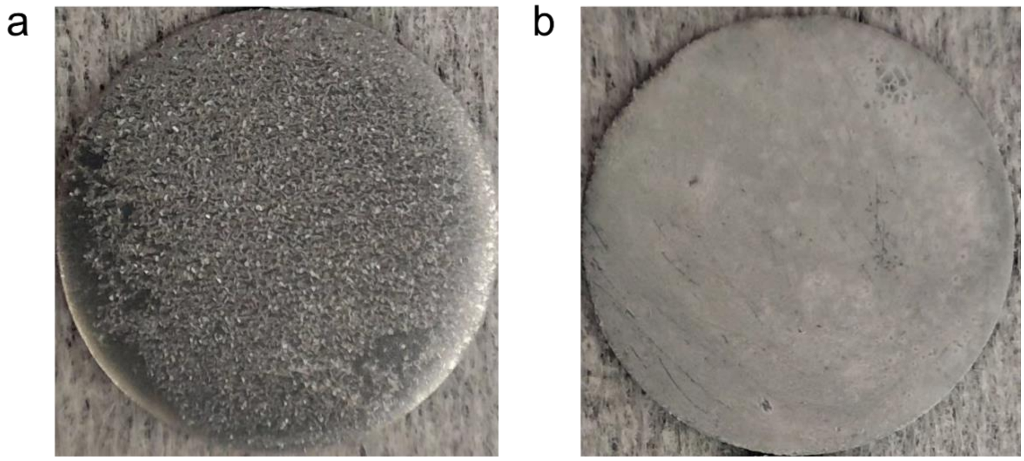

Supplementary Fig. S8 | The optical photos of zinc foils soaked for 15 days in **a** the conventional electrolyte; **b** the densified electrolyte.

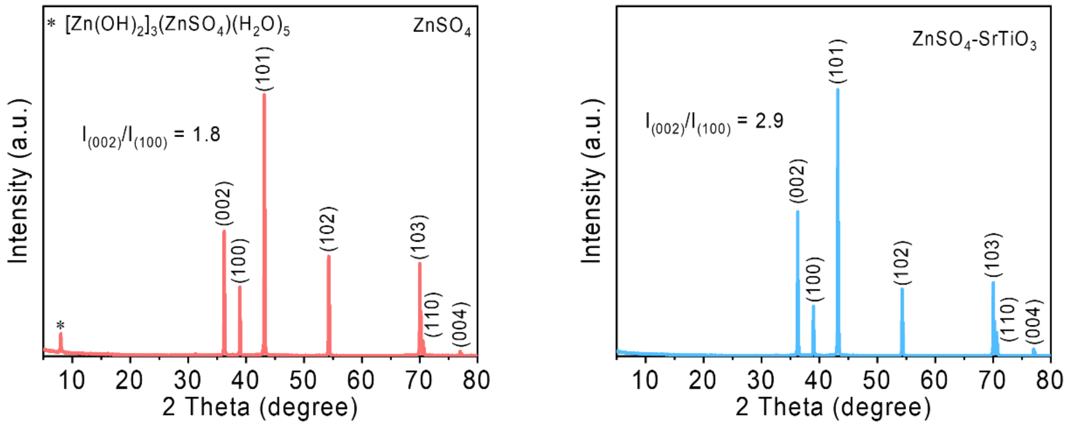

Supplementary Fig. S9 | The XRD patterns of the zinc foils after 10 h deposition in various electrolytes.

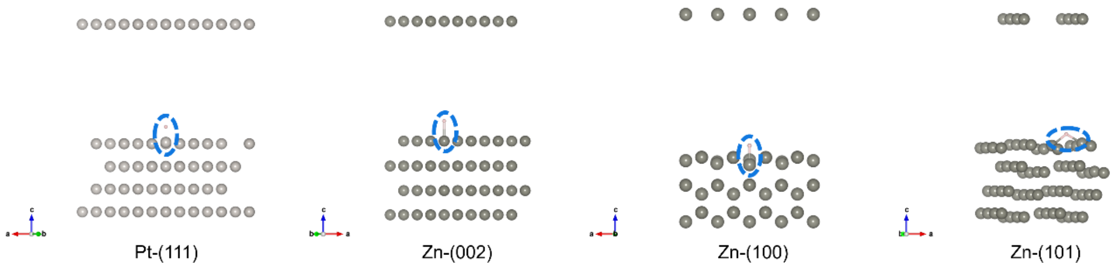

Supplementary Fig. S10 | The geometrical configurations of the H adsorption energy.

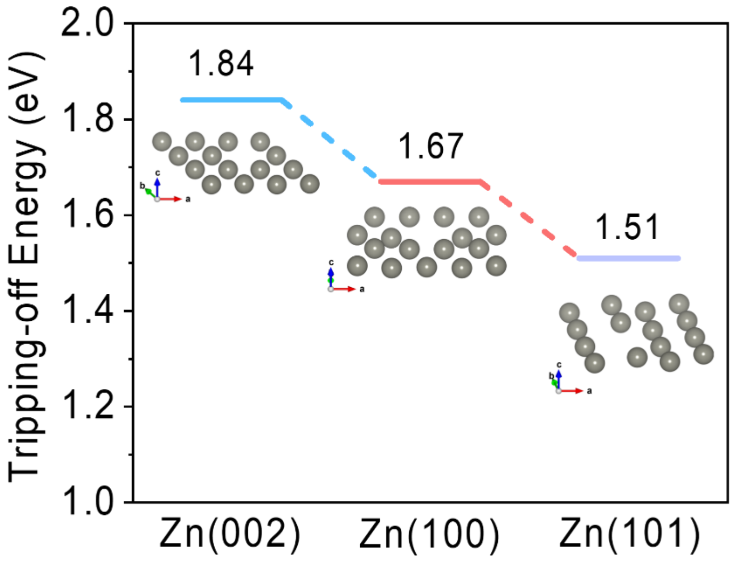

Supplementary Fig. S11 | The Zn tripping off energy at various zinc crystal planes.

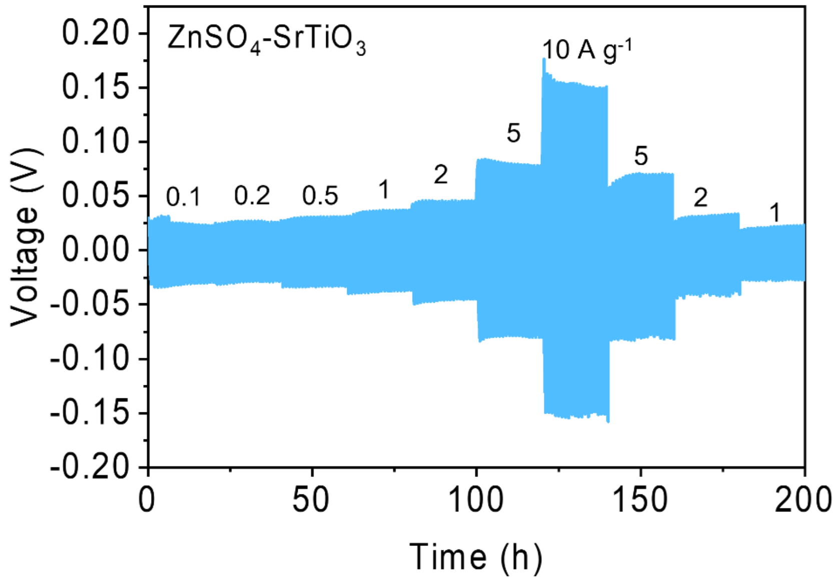

Supplementary Fig. S12 | The rate performance of Zn/Zn symmetric cells using densified electrolyte with various current densities of 0.1, 0.2, 0.5, 1, 2, 5, 10 A g^{-1}.

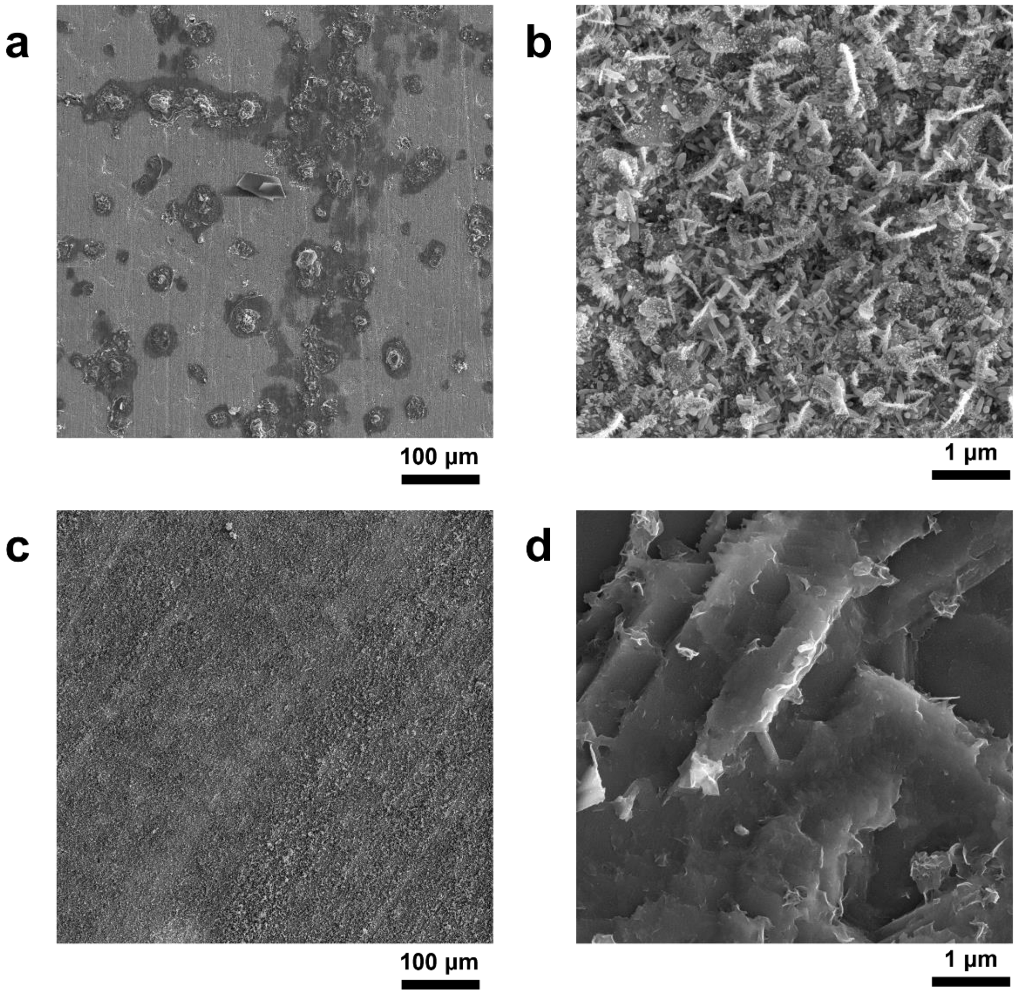

Supplementary Fig. S13 | The SEM images of Zn anodes obtained from the Zn/Zn symmetric cells cycled 50 hours at 1 mA cm^{-2}. **a, b** in conventional electrolyte; **c, d** in densified electrolyte.

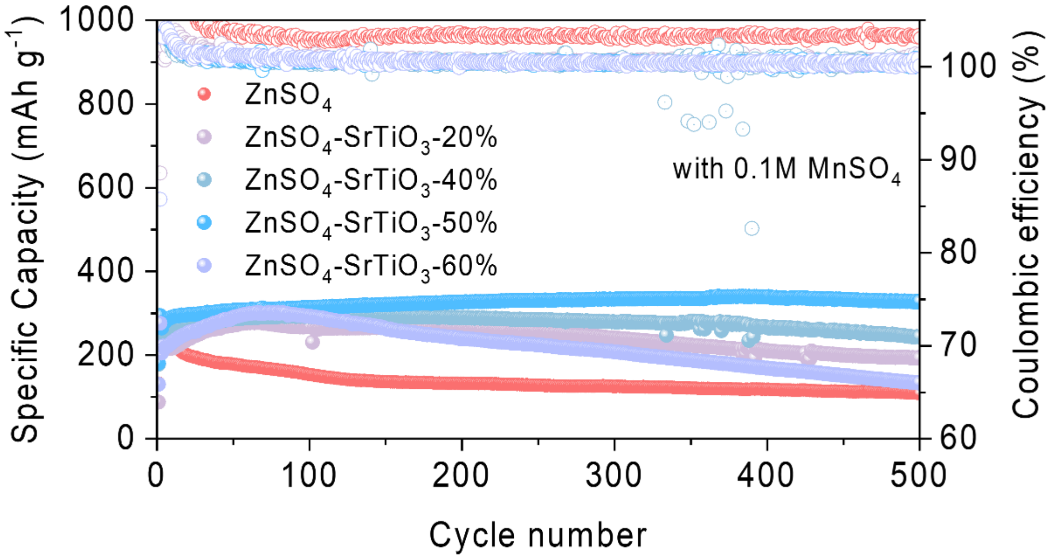

Supplementary Fig. S14 | The long-term galvanostatic cycling performance of Zn/MnO_2 cells

in electrolytes with different $SrTiO_3$ contents at a current density of 1 A g^{-1}.

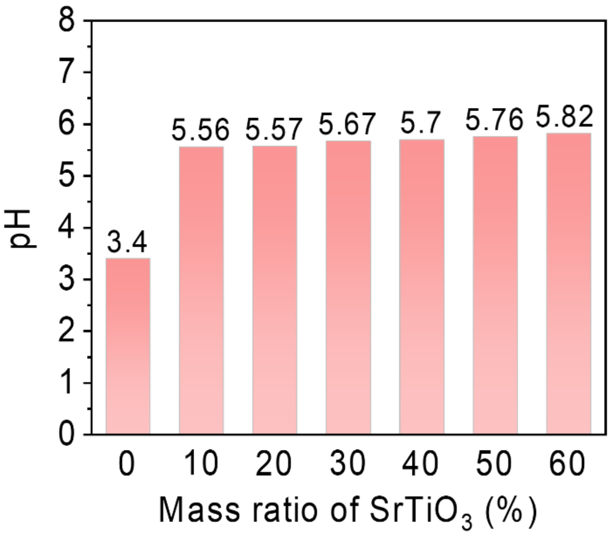

Supplementary Fig. S15 | The pH of conventional electrolyte and densified electrolytes with different $SrTiO_3$ contents.

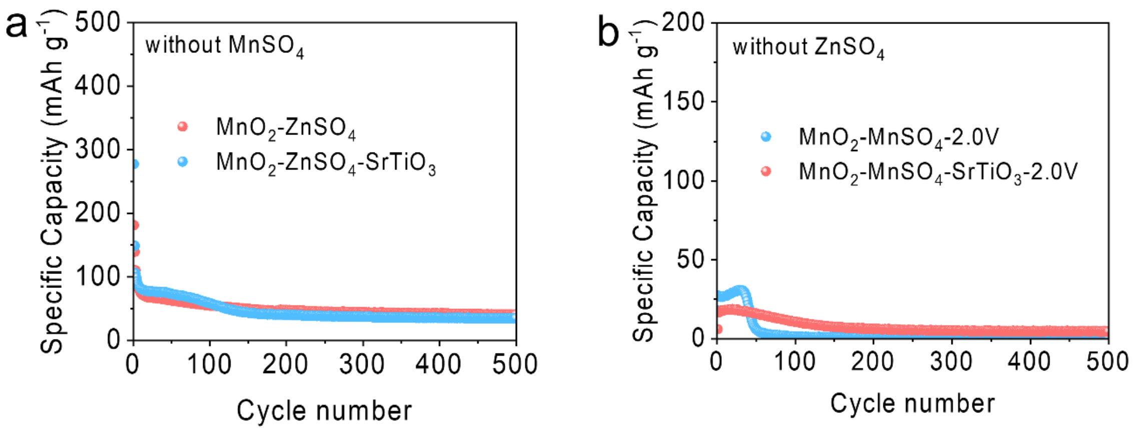

Supplementary Fig. S16 | The galvanostatic cycling performance of Zn/MnO_2 cells at a current density of 1 A g^{-1} in various electrolytes: **a** without $MnSO_4$ and **b** without $ZnSO_4$.

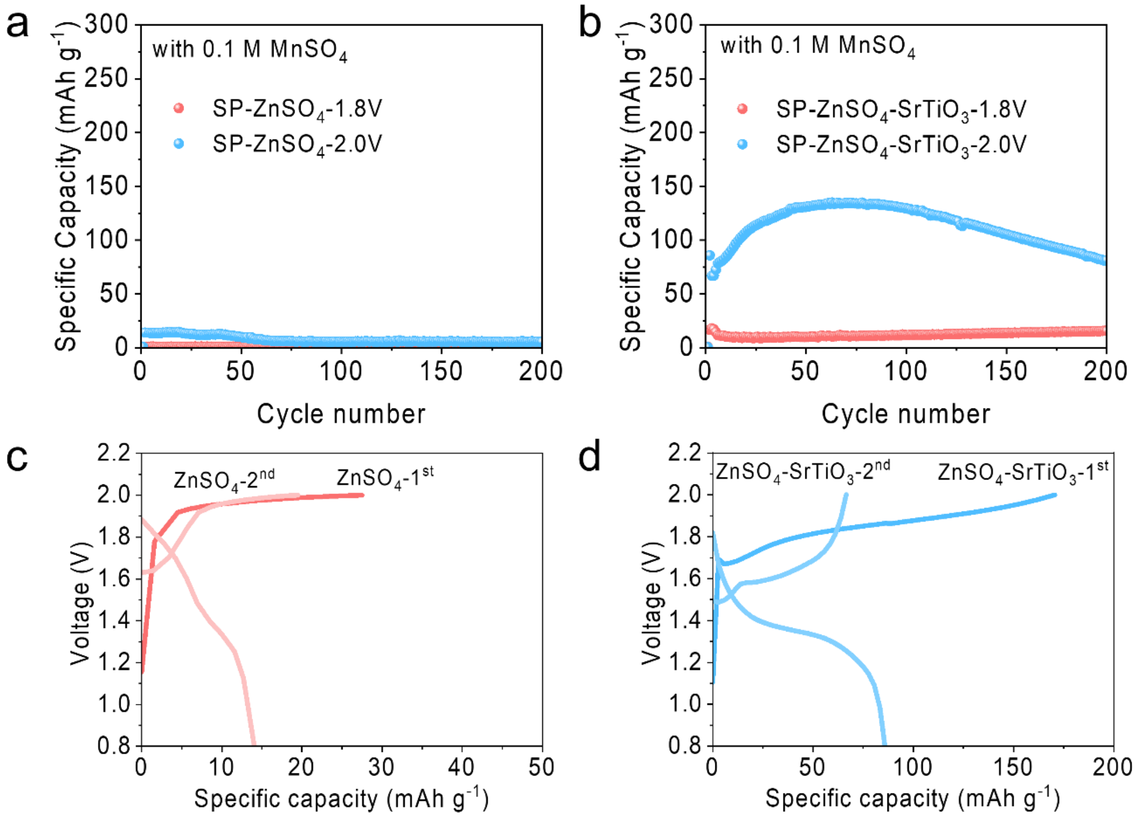

Supplementary Fig. S17 | The galvanostatic cycling performance of Zn/SP cells (set the active material mass is 1 mg) under different charge cut-off voltage at a current density of 1 A g^{-1} in **a** conventional electrolytes and **b** densified electrolyte; The first two charge-discharge curves of SP cells under charge cut-off voltage of 2.0 V in **c** conventional electrolytes and **d** densified electrolytes.

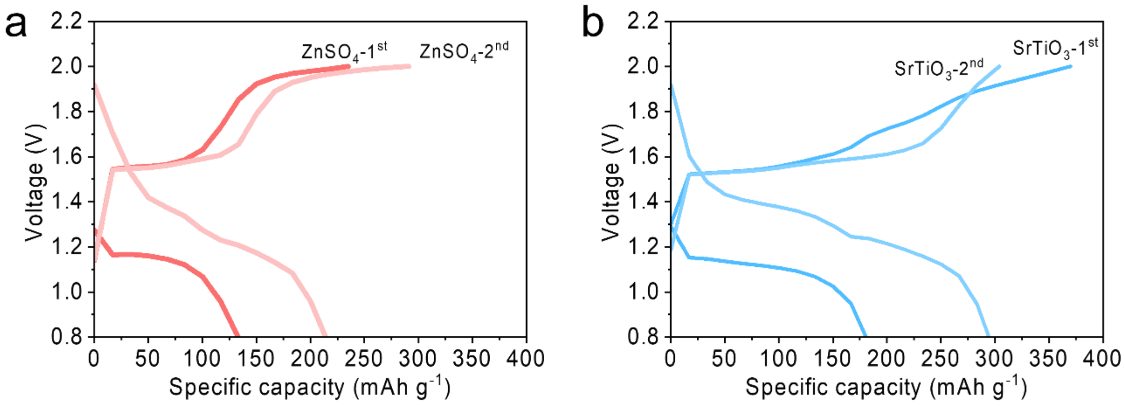

Supplementary Fig. S18 | The first two charge and discharge curves of Zn/MnO_2 full cells in **a** conventional electrolyte and **b** densified electrolyte.

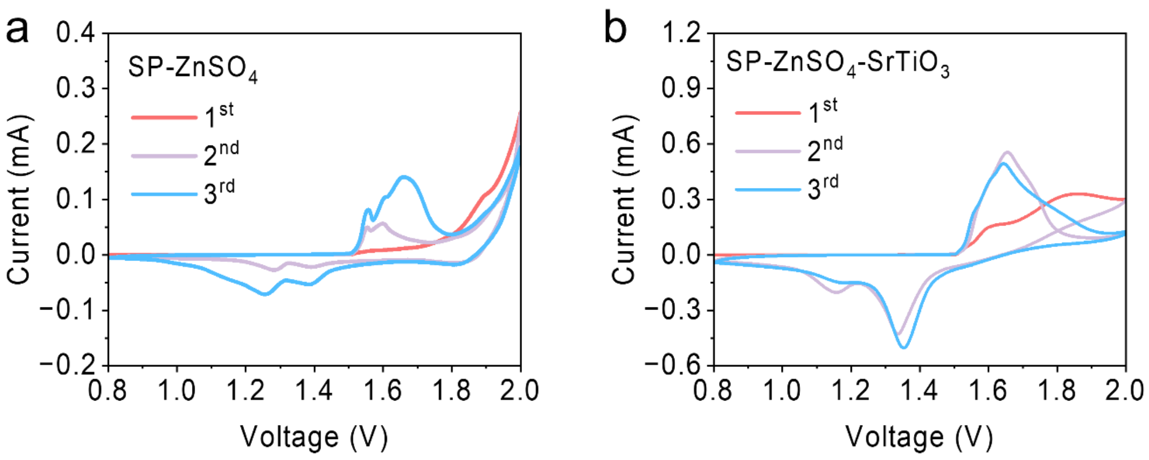

Supplementary Fig. S19 | The cyclic voltammetry (CV) profiles of SP cells at a scan rate of 0.1 mV s^{-1}: **a** in the conventional electrolyte and **b** in the densified electrolyte.

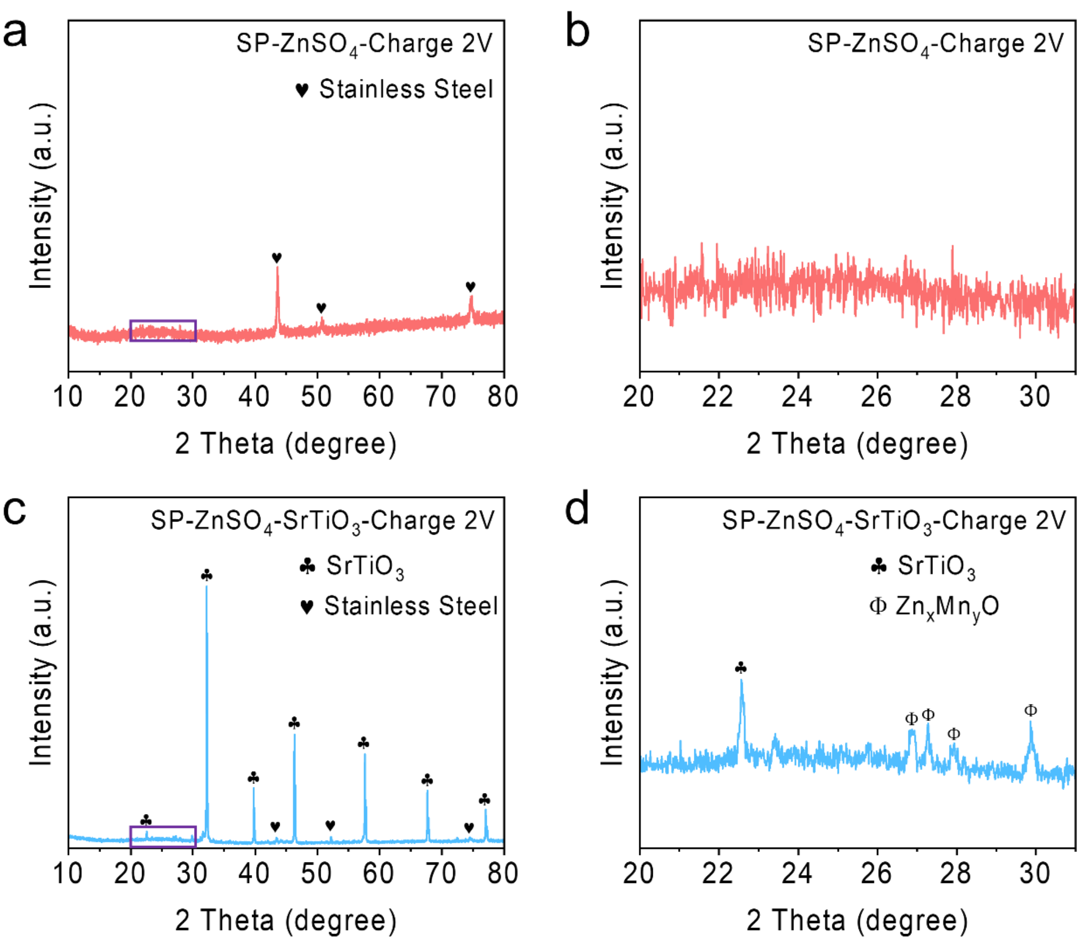

Supplementary Fig. S20 | The investigation on the redox behavior of the first charge to 2.0 V: **a** the XRD pattern and **b** the corresponding local magnification ranges (marked area) of the SP cathode charged in the conventional electrolyte; **c** the XRD pattern and **d** the corresponding local magnification ranges (marked area) of the SP cathode charged in the densified electrolyte.

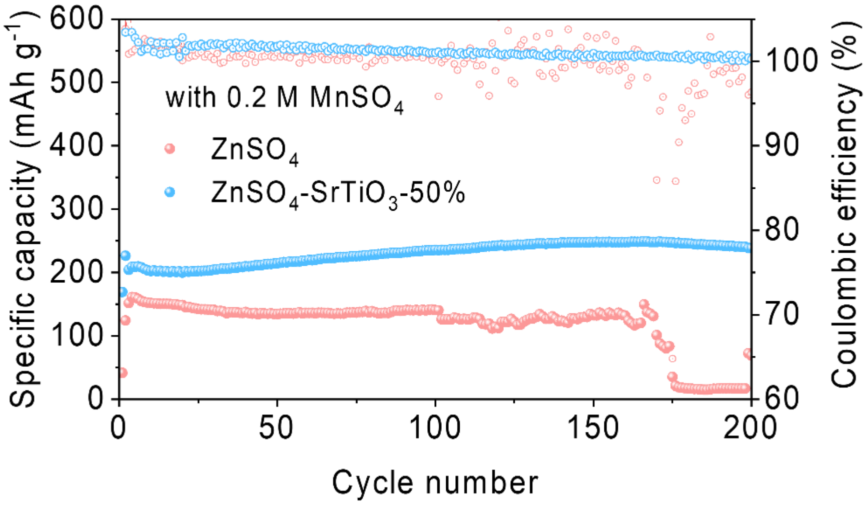

Supplementary Fig. S21 | The galvanostatic cycling performance of Zn/MnO_2 cells with a higher-loading of 4 mg cm^{-2} in various electrolytes at a current density of 0.5 A g^{-1}.

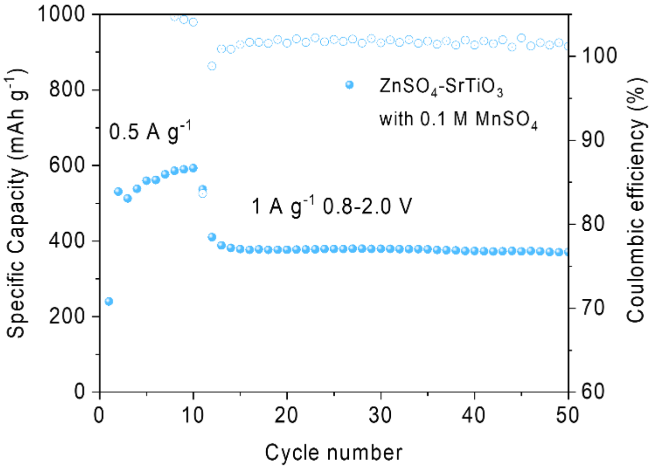

Supplementary Fig. S22 | The electrochemical performance of the cell using densified electrolyte after ten cycles of activation at 0.5 A g^{-1}.

The higher specific capacity may be due to the presence of an activation process, which leads to more zinc-manganese oxide generation and thus contributes more specific capacity (**Supplementary Fig. S22**). And this is the reason why the specific capacity of the cell using densified electrolyte recovers to 392.6 mAh g^{-1}, which is higher than that of the cell before rate tests and the cells in **Fig. 6a** that cycle directly at 1A g^{-1}, when the applied current density returns to 1 A g^{-1}.

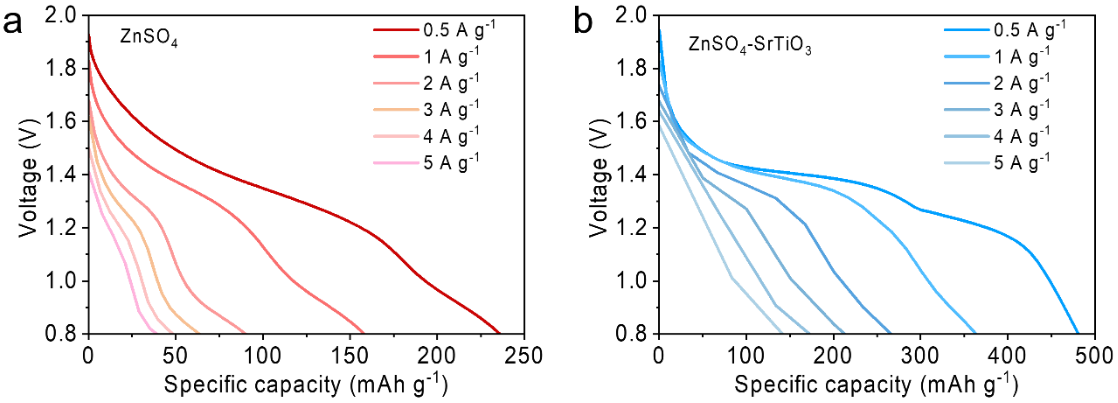

Supplementary Fig. S23 | The discharge curves of Zn/MnO_2 full cell at different rate. **a** in conventional electrolyte; **b** in densified electrolyte.

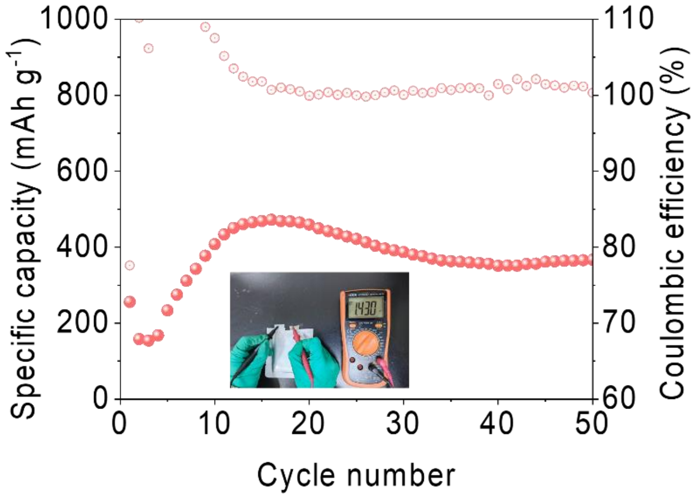

Supplementary Fig. S24 | The long cycling performance of Zn/MnO_2 pouch cells (the inset picture shows the open-circuit voltage of the pouch cell).

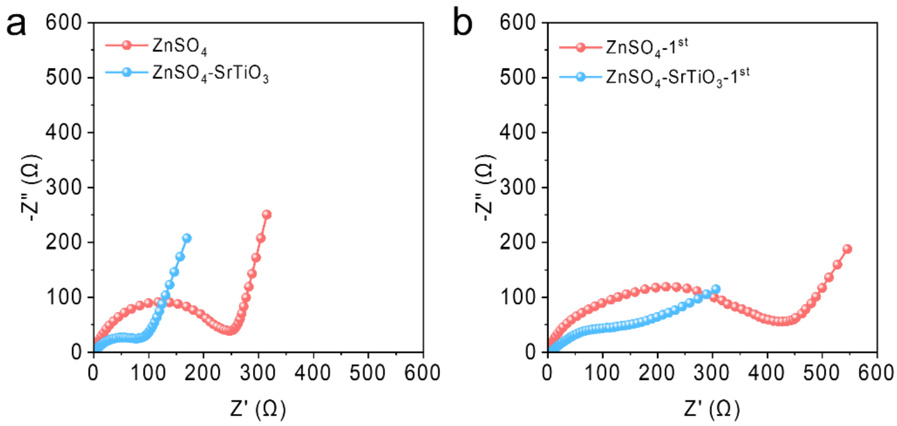

Supplementary Fig. S25 | The EIS of full cells using various electrolytes: **a** before cycle and **b** after first cycle.

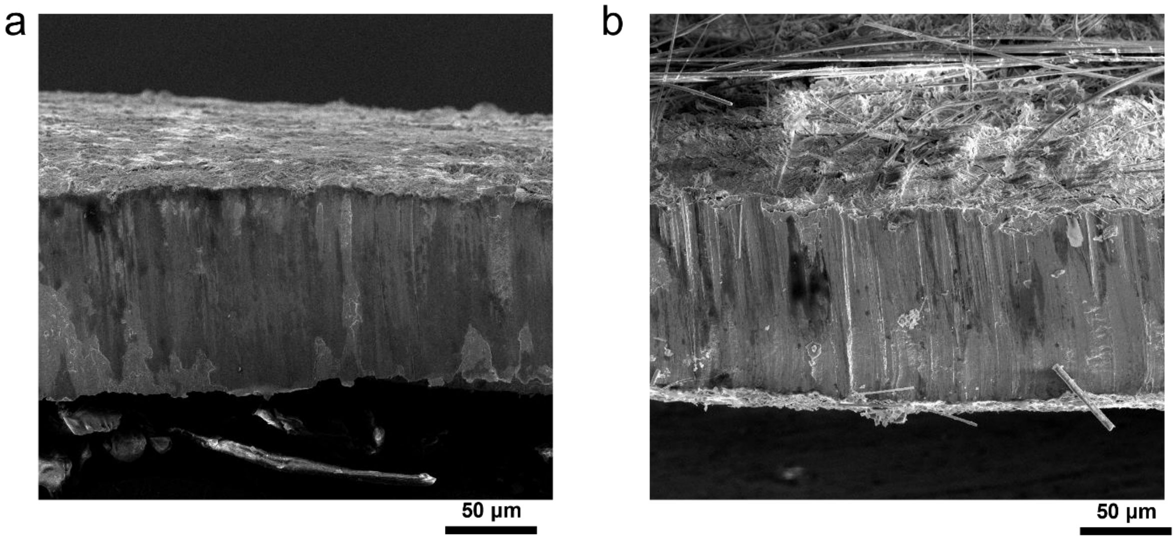

Supplementary Fig. S26 | The cross-sectional images of zinc anodes after 500 cycles from the full cells using **a** conventional electrolyte; **b** densified electrolyte.
