# Supplementary material for: An aqueous electrolyte densified by perovskite SrTiO3 enabling high-voltage zinc-ion batteries
Source: Nat Commun. 2023 Aug 17;14:4981. doi: 10.1038/s41467-023-40462-z (PMC10435537; doi:10.1038/s41467-023-40462-z)
Supplement: Supplementary file 3 — Reporting Summary [file 41467_2023_40462_MOESM3_ESM.pdf]

nature portfolio

Corresponding author(s): Feixiang Wu

Last updated by author(s): Jul 13, 2023

Reporting Summary

Nature Portfolio wishes to improve the reproducibility of the work that we publish. This form provides structure for consistency and transparency in reporting. For further information on Nature Portfolio policies, see our Editorial Policies and the Editorial Policy Checklist.

Statistics

For all statistical analyses, confirm that the following items are present in the figure legend, table legend, main text, or Methods section.

n/a	Confirmed	
x		The exact sample size (*n*) for each experimental group/condition, given as a discrete number and unit of measurement
	x	A statement on whether measurements were taken from distinct samples or whether the same sample was measured repeatedly
x		The statistical test(s) used AND whether they are one- or two-sided<br>*Only common tests should be described solely by name; describe more complex techniques in the Methods section.*
x		A description of all covariates tested
	x	A description of any assumptions or corrections, such as tests of normality and adjustment for multiple comparisons
	x	A full description of the statistical parameters including central tendency (e.g. means) or other basic estimates (e.g. regression coefficient) AND variation (e.g. standard deviation) or associated estimates of uncertainty (e.g. confidence intervals)
x		For null hypothesis testing, the test statistic (e.g. *F*, *t*, *r*) with confidence intervals, effect sizes, degrees of freedom and *P* value noted<br>*Give P values as exact values whenever suitable.*
x		For Bayesian analysis, information on the choice of priors and Markov chain Monte Carlo settings
x		For hierarchical and complex designs, identification of the appropriate level for tests and full reporting of outcomes
x		Estimates of effect sizes (e.g. Cohen's *d*, Pearson's *r*), indicating how they were calculated

Our web collection on statistics for biologists contains articles on many of the points above.

Software and code

Policy information about availability of computer code

Data collection	VESTA VERSION 3.5.3 was used to generate and edit crystal structure.
Data analysis	see above.

For manuscripts utilizing custom algorithms or software that are central to the research but not yet described in published literature, software must be made available to editors and reviewers. We strongly encourage code deposition in a community repository (e.g. GitHub). See the Nature Portfolio guidelines for submitting code & software for further information.

Data

Policy information about availability of data

All manuscripts must include a data availability statement. This statement should provide the following information, where applicable:

- Accession codes, unique identifiers, or web links for publicly available datasets
- A description of any restrictions on data availability
- For clinical datasets or third party data, please ensure that the statement adheres to our policy

The authors declare that the data supporting the findings of this study are available within the paper and its Supplementary Information files. Should any raw data files be needed in another format they are available from the corresponding author upon reasonable request. Source data are provided with this paper.

Research involving human participants, their data, or biological material

Policy information about studies with human participants or human data. See also policy information about sex, gender (identity/presentation), and sexual orientation and race, ethnicity and racism.

Reporting on sex and gender	All experiments involved chemical and electrochemical analysis and do not involve any sex and gender.
Reporting on race, ethnicity, or other socially relevant groupings	Not involved in this study.
Population characteristics	Not involved in this study.
Recruitment	Not involved in this study.
Ethics oversight	Not involved in this study.

Note that full information on the approval of the study protocol must also be provided in the manuscript.

Field-specific reporting

Please select the one below that is the best fit for your research. If you are not sure, read the appropriate sections before making your selection.

☐ Life sciences ☐ Behavioural & social sciences ☒ Ecological, evolutionary & environmental sciences

For a reference copy of the document with all sections, see nature.com/documents/nr-reporting-summary-flat.pdf

Ecological, evolutionary & environmental sciences study design

All studies must disclose on these points even when the disclosure is negative.

Study description	Study on the electrolyte design for zinc-ion batteries, this study constructs an aqueous densified electrolyte with reduced water molecular activity and high cation transference number using the metal oxide SrTiO3 as an additive.
Research sample	The main samples like ZnSO4·7H2O, and SrTiO3 were purchased from Aladdin Reagent (Shanghai) Co., Ltd<br>The prepared MnO2 is the cathode material.
Sampling strategy	Strontium titanate particles are less than 5 microns in size
Data collection	Rongyu Deng recorded the main data by using battey test LAND systems. All data were collected under appropriate test conditions.
Timing and spatial scale	The data were collected from May 6, 2022 to January 30, 2023 in Changsha, China.
Data exclusions	No data exclusions.
Reproducibility	Ensure that no impurities are introduced.
Randomization	1. The prepared MnO2 were grouped wherether therer was modification. The electrolytes were grouped based om the salt, concentration, and additives.<br>2. Electrodes were grouped according to which electrolyte they cycled in.<br>3. For micro areas on the electrode, no grouping.
Blinding	Blinding was not involved in this study.

Did the study involve field work? ☐ Yes ☒ No

Reporting for specific materials, systems and methods

We require information from authors about some types of materials, experimental systems and methods used in many studies. Here, indicate whether each material, system or method listed is relevant to your study. If you are not sure if a list item applies to your research, read the appropriate section before selecting a response.

Materials & experimental systems

n/a	Involved in the study
☒	☐ Antibodies
☒	☐ Eukaryotic cell lines
☒	☐ Palaeontology and archaeology
☒	☐ Animals and other organisms
☒	☐ Clinical data
☒	☐ Dual use research of concern
☒	☐ Plants

Methods

n/a	Involved in the study
☒	☐ ChIP-seq
☒	☐ Flow cytometry
☒	☐ MRI-based neuroimaging
